# When Nothing Else Works: Fresh Frozen Plasma in the Treatment of Progressive, Refractory Angiotensin-Converting Enzyme Inhibitor–Induced Angioedema

**DOI:** 10.7759/cureus.972

**Published:** 2017-01-11

**Authors:** Gerard Chaaya, Faraz Afridi, Arfa Faiz, Ali Ashraf, Mahrukh Ali, Analia Castiglioni

**Affiliations:** 1 Internal Medicine, University of Central Florida College of Medicine; 2 Pulmonary/Critical Care, Osceola Regional Medical Center

**Keywords:** fresh frozen plasma, angioedema, angiotensin-converting enzyme inhibitor

## Abstract

Angioedema is a severe form of an allergic reaction characterized by the localized edematous swelling of the dermis and subcutaneous tissues. Angiotensin-converting enzyme inhibitor-induced angioedema (ACEI-IA) is an allergic reaction that can be severe in some cases requiring advanced management measures. Fresh frozen plasma has been used off-labeled in some case reports to improve and to prevent worsening of the angioedema in a few cases of ACEI-IA. We are reporting this case to increase the awareness of physicians and to widen their therapeutic options when encountering this clinically significant condition.

## Introduction

Angioedema (AE) is a severe form of an allergic reaction characterized by the localized edematous swelling of the dermis and subcutaneous tissues, usually involving the head and neck area (face, lips, tongue, larynx, pharynx). Angiotensin-converting enzyme inhibitors (ACEIs) are commonly prescribed cardiovascular medications known to cause angioedema in some patients [1].

## Case presentation

**Figure 1:**
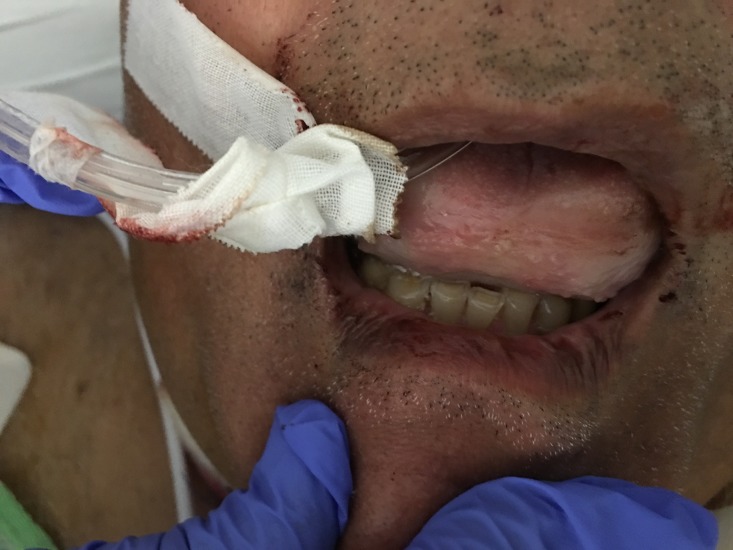
Markedly swollen tongue obscuring the entire soft palate

A 66-year-old Asian male presented to the emergency department with the acute onset of tongue swelling after taking his usual dose of lisinopril that evening. His past medical history was significant for hypertension, hyperlipidemia, diastolic congestive heart failure, rheumatoid arthritis, and Stevens-Johnson syndrome secondary to allopurinol and penicillin use in the past. The patient had been on lisinopril for almost a year. Other home medications included carvedilol, hydrochlorothiazide, simvastatin, aspirin, methotrexate, and folic acid. The patient denied constitutional symptoms, sick contacts, and recent travel. On presentation, physical exam, including vital signs, were normal except for a loss of phonation and a markedly swollen tongue, obscuring the entire soft palate, with a Mallampati Class IV (Figure 1).

Complete blood count, comprehensive metabolic panel, coagulation studies, C3, C4, total complement level (CH50), and C1 esterase inhibitor were within normal limits.

**Figure 2:**
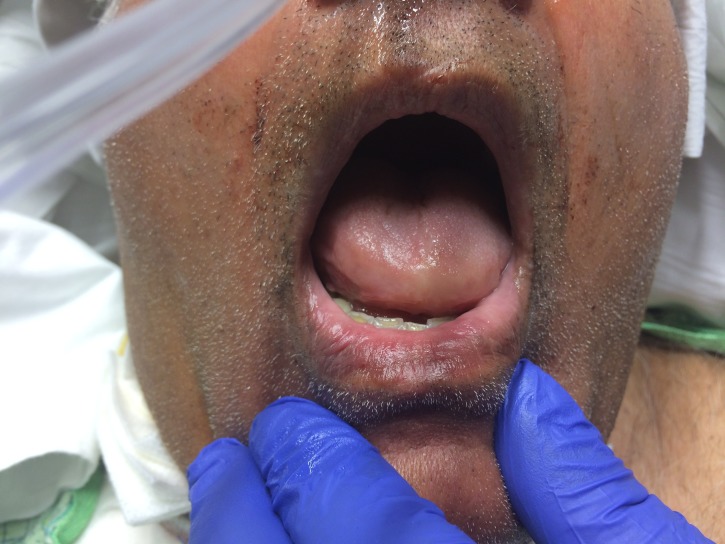
Complete resolution of the swelling within four hours of FFP administration FFP: fresh frozen plasma

In the emergency department, besides discontinuing lisinopril, the patient received 0.3 mg of epinephrine (1:1000) subcutaneously, 125 mg of methylprednisolone intravenously (IV), 50 mg of diphenhydramine IV, and 20 mg of famotidine IV, all with no improvement in status. Informed patient consent was waived due to the emergent condition of the patient. A fiberoptic nasal intubation for airway protection was unsuccessful because of the edematous oropharynx; thus, a cricothyroidotomy was performed. The patient desaturated after the procedure, requiring evaluation in the operating room, which led to emergent tracheostomy tube placement. Once stabilized, the patient was transferred to the intensive care unit. Given the persistent nature of his angioedema, he was administered two units of fresh frozen plasma (FFP) in an attempt to lessen the severity of angioedema. Almost complete resolution of the swelling was observed within four hours of FFP administration (Figure 2).

The patient was decannulated two days later and was discharged on a calcium channel blocker instead of an angiotensin-converting enzyme inhibitor (ACEI).

## Discussion

The mechanism of developing ACE inhibitor-induced angioedema (ACEI-IAE) is due to the ACE inhibition, which accumulates bradykinin (a potent vasodilator) and leads to increased capillary and vascular permeability producing the characteristic swelling [2].

ACEI-IAE carries a high risk of morbidity by causing airway compromise and respiratory distress. There are no specific guidelines or algorithms for the management of ACEI-IA, and clinical trials have been limited [3]. Current management includes immediate ACEI discontinuation and the initiation of antihistamines, anticholinergics, steroids, and epinephrine [4]. Several new drugs, such as a recombinant C1 esterase inhibitor (C1-INH), a kallikrein inhibitor (ecallantide), and a specific bradykinin-B2-receptor antagonist (icatibant), were developed for the treatment of ACEI-IAE but have not been widely available for clinical use [4]. Angioedema caused by ACEIs, in the majority of cases, usually resolves within 24 to 72 hours [5].

Off-label use of FFP has been described for reversing the effects of ACEI-IAE, especially in patients who have life-threatening symptoms (dyspnea, dysphonia, odynophagia, stridor, drooling, or respiratory distress) or for refractory cases not responding to ACEI discontinuation and standard therapies [2, 6]. FFP contains kininase II (identical to ACE) that leads to the degradation of bradykinin. Case reports have described administration of FFP leading to rapid improvement of ACE-IAE without further recurrence of symptoms [1]. The current recommendations are the use of two units of FFPs for adults, and the swelling has been documented to resolve within two to four hours of administration [4]. Risks with the use of FFP include potential for initial worsening of angioedema (specifically in hereditary angioedema due to its bradykinin content), viral transmission, delayed administration time, allergic/transfusion reactions, and volume overload [4].

## Conclusions

Our case illustrates an example of the successful use of FFP for the treatment of life-threatening ACE-IAE refractory to conventional therapy. Physicians treating patients presenting with severe ACE-IAE should keep this treatment in mind when steroids, antihistamines, and epinephrine fail to reverse symptoms. In the light of the fast response to FFP observed in our patient, it is possible that an earlier administration of FFP, immediately after conventional therapy failure was recognized, could have prevented the need for invasive airway management.
